# Text intelligent correction in English translation: A study on integrating models with dependency attention mechanism

**DOI:** 10.1371/journal.pone.0319690

**Published:** 2025-06-24

**Authors:** Yutong Liu, Shile Zhang

**Affiliations:** 1 School of Humanities and Social Sciences, Xi’an Polytechnic University, Xi’an, China; 2 Shaanxi Contemporary Red Culture Training and Education Center, Xi’an, China; SR University, INDIA

## Abstract

Improving translation quality and efficiency is one of the key challenges in the field of Natural Language Processing (NLP). This study proposes an enhanced model based on Bidirectional Encoder Representations from Transformers (BERT), combined with a dependency self-attention mechanism, to automatically detect and correct textual errors in the translation process. The model aims to strengthen the understanding of sentence structure, thereby improving both the accuracy and efficiency of error correction. The research uses the Conference on Natural Language Learning (CoNLL)-2014 dataset as an experimental benchmark, which contains a rich collection of grammatical error samples and is a standard resource in linguistic research. During model training, the Adam optimization algorithm is employed, and the model’s performance is enhanced by introducing a customized dependency self-attention mechanism for parameter optimization. To validate the model’s effectiveness, the performance of the baseline model and the improved model is compared using multiple evaluation metrics, including accuracy, recall, F1 score, edit distance, Bilingual Evaluation Understudy (BLEU) score, and average processing time. The results show that the proposed model significantly outperforms the baseline model in terms of accuracy (improving from 0.78 to 0.85), recall (improving from 0.81 to 0.87), and F1 score (improving from 0.79 to 0.86). The average edit distance decreases from 3.2 to 2.5, the BLEU score increases from 0.65 to 0.72, and the average processing time is reduced from 2.3 seconds to 1.8 seconds. This study provides an innovative approach for intelligent text correction tasks, expands the application scenarios of the BERT model, and offers significant support for the practical implementation of NLP technologies. The findings not only highlight the advantages of the improved model but also offer new ideas and directions for future related research.

## Introduction

In today’s globalized context, the importance of English as a universal language has become increasingly prominent, with its widespread application across international communication, multinational business activities, and academic research [1]. The proliferation of English has not only facilitated the dissemination of culture and knowledge but also laid the foundation for international cooperation and commercial prosperity. However, with the rapid growth in the number of non-native English learners, the complexity and diversity of English in practical use have posed numerous challenges [2,3]. For users who speak English as a second language or foreign language, both written and spoken communication can be severely impacted by grammatical errors, improper vocabulary choices, or even semantic ambiguity due to limited language proficiency or cultural background differences. This issue is especially evident in the translation process, particularly in business and professional fields. Even slight grammatical errors, vocabulary misuse, or punctuation mistakes can lead to misunderstandings or ambiguity, which can negatively affect communication effectiveness, project execution efficiency, and the outcomes of business negotiations [4,5]. For instance, language errors in contract terms, legal documents, or technical reports not only have the potential to cause financial losses but may also harm the trust relationships between stakeholders.

To address these challenges, text intelligent error correction has become a key research direction in the field of Natural Language Processing (NLP) and has garnered widespread attention in recent years. Traditional English text correction methods primarily rely on rule-based or statistical models, which identify and correct text errors by predefined language rules, vocabulary glossaries, or statistical probabilities. These methods perform well when handling simple grammatical errors (such as tense consistency or singular/plural changes), but they struggle to achieve satisfactory results when dealing with complex grammatical structures or errors that heavily depend on context [6]. For example, when faced with long sentences, nested clauses, or situations requiring the integration of multiple layers of semantic information, rule-based and statistical methods often fall short due to their rigid nature or lack of contextual modeling ability. With the rapid development of deep learning technologies, the use of neural network models for intelligent text error correction has gradually become a research focus. Deep learning-based correction methods, with their powerful feature extraction and learning capabilities, can capture complex linguistic phenomena and effectively model contextual dependencies. Especially with the advent of technologies like the Transformer architecture and pre-trained language models, deep learning has provided new solutions to improve correction efficiency and accuracy [7]. These methods not only handle complex grammatical errors but also adapt to the practical application needs of multiple languages and contexts. They achieve this by continually optimizing model structures and training algorithms, thereby advancing language technology and providing strong technical support for cross-cultural communication.

In the field of text error correction, many researchers have explored various approaches. An overview of the related work is shown in Table 1.

**Table 1:** Overview of Related Work.

Researcher	Research Focus	Main Method	Main Contribution	Experimental Results
Yang (2024) [8]	Automatic error correction in business English texts	Triplet conversion based on NLP, Maximum Likelihood Estimation, constructing Naive Bayes classifier, beam search decoding	Proposed a method for automatic error correction in business English texts	Good performance in translation error correction accuracy, average error, and error correction time
Wang et al. (2023) [9]	Chinese text error correction model	Fusion of Chinese character knowledge graph and Bidirectional Encoder Representations from Transformers (BERT) model, integrating pronunciation, character form, and semantic features	Proposed a Chinese text error correction model for the security field	Outperformed other models in security domain text correction tasks
Najam and Faizullah (2023) [10]	Arabic handwritten text OCR (Optical Character Recognition) and text correction	Using Convolutional Neural Network - Long Short-Term Memory - Connectionist Temporal Classification (CNN-LSTM-CTC) architecture for optical character recognition	Improved accuracy of Arabic OCR text correction	Deep learning models significantly improved the accuracy of OCR text correction
Priya et al. (2022) [11]	Spelling correction in speech recognition systems	BERT-based spelling correction module	Improved accuracy of speech recognition systems	BERT-based spelling correction significantly improved recognition accuracy across different datasets
Wang and Zhong (2022) [12]	English grammar detection model	Used deep learning techniques to build the Automatic Syntax and Spelling (ASS) model for grammar correction	Proposed a model that improves grammar and spelling correction accuracy	Overall accuracy improved by 54.81%, with the model achieving 98.71% accuracy
Li et al. (2022) [13]	Grammar error correction method based on syntax trees	Introduced a grammar graph combining dependency trees and constituent trees, using graph encoders and cross-attention mechanisms for sentence encoding	Proposed a grammar error correction method combining graph encoders and cross-attention mechanisms	Validated the model’s effectiveness on the CoNLL-2014 and JFLEG-GEC benchmarks
Koneru et al. (2023) [14]	Application of Large Language Models (LLMs) in translation tasks	Fine-tuned large language models using low-rank adapters for automatic post-editing	Proposed applying large language models to automatic post-editing for translation optimization	Achieved 89% accuracy on the ContraPro test set, with significant document-level translation improvements
Ki & Carpuat (2024) [15]	Guiding large language models for machine translation post-editing	Used external quality feedback and fine-tuning strategies to guide large language models in post-editing, combining the strengths of machine translation	Improved large language models’ performance in machine translation post-editing	Enhanced translation; Translation Edit Rate (TER), BLEU score, and Crosslingual Optimized Metric for Evaluation of Translation (COMET) score through fine-tuning

Existing studies, such as BERT in bidirectional semantic understanding and Transformer in modeling long-distance dependencies, have achieved significant results. However, these methods primarily focus on the pretraining and fine-tuning capabilities of general language models, with limited attention to the dependency relationships and syntactic structures of the input text. Additionally, although existing dependency parsers can capture syntactic dependencies, their error correction abilities remain notably insufficient. To address these shortcomings, this study proposes a sequence error correction model based on dependency self-attention, which integrates dependency distance information into the self-attention mechanism. A Transformer model with dependency self-attention is constructed, aiming to improve both syntactic understanding and error correction performance simultaneously. Through improvements to existing parsers and models, this study offers a more efficient error correction solution and expands the integration of dependency syntactic analysis with sequence-to-sequence models.

The novelty of this study lies in several key aspects: (1) This study applies the dependency self-attention mechanism to the field of intelligent text error correction, enhancing the model’s ability to understand grammar and syntactic structure. Compared to traditional context-based attention mechanisms, the dependency self-attention mechanism more accurately captures the dependency structure between words in a sentence by incorporating syntactic dependencies, thus improving the accuracy and efficiency of error correction. (2) Based on the BERT model, this study proposes an integrated framework that incorporates the dependency self-attention mechanism during the pretraining phase and further optimizes model performance during the fine-tuning phase. This method retains BERT’s advantages in semantic understanding while enhancing the effectiveness of the text error correction task through structured optimization, enabling precise identification and efficient correction of erroneous text. (3) Through a series of detailed ablation experiments, this study systematically analyzes the contribution of key modules, such as pretraining, fine-tuning, and the dependency self-attention mechanism, to model performance, clarifying the importance of each part of the model. This in-depth analysis not only validates the effectiveness of the method but also provides references and insights for future research. (4) In the experimental evaluation, this study uses a range of multidimensional metrics (such as accuracy, recall, F1 score, average edit distance, BLEU score, and average processing time) to conduct a comprehensive assessment of model performance. This evaluation approach allows for a more nuanced presentation of the model’s performance across different aspects, providing strong empirical support for the superiority of the method. (5) This study focuses on the task of intelligent text error correction in English translation, an area with a high demand for efficient and accurate text processing. Through the application of innovative methods, this study provides a feasible solution to the text error correction task, not only improving correction efficiency and accuracy but also offering new ideas and tools for future research and practical applications. Therefore, this study combines the latest NLP technologies with in-depth experimental analysis. It not only fills certain gaps in current research but also provides valuable references for the further development of the text error correction field with practical significance.

The main contributions of this study include: (1) Introduction of the Dependency Self-Attention Mechanism: By incorporating dependency distance into the self-attention mechanism, this study proposes a sequence-to-sequence text error correction model based on dependency information, which significantly enhances the model’s ability to understand and correct grammatical errors. (2) Improvement of the Dependency Parser: Building upon existing parsers, a new dependency parser designed specifically for syntactically incorrect sentences is introduced. This parser enables the extraction of valid dependency information from erroneous sentences. (3) Comprehensive Performance Improvement: Experimental validation shows that the proposed model outperforms existing baseline models and advanced models in key metrics such as accuracy, recall, F1 score, and BLEU score. It also demonstrates higher efficiency in terms of average processing time. (4) Ablation Experiments and Performance Evaluation: Detailed ablation experiments were conducted to quantify the contributions of various model components, further validating the effectiveness and robustness of the dependency self-attention mechanism. (5) Optimization for Practical Applications: For scenarios with limited computational resources, the proposed model offers a superior, efficient, and practical text error correction solution.

The structure of this study is divided into the following sections: (1) Introduction: The introduction presents the research background and significance, clearly defines the research problem related to intelligent text error correction, and outlines the research methods and innovations. A review of the related theories and methods in the field of intelligent error correction is provided, with a focus on the BERT framework and its application in NLP tasks. The potential of the dependency self-attention mechanism to enhance language modeling capabilities is also discussed. (2) Model Design: This study proposes an integrated model that combines the dependency self-attention mechanism. The design details include pretraining, fine-tuning, and the construction and integration of the dependency self-attention module. The model implementation process and key technical points are explained, and the experimental environment, along with the dataset selection and processing methods, are introduced. (3) Experimental Data Design. (4) Experimental Validation and Result Analysis: The model’s performance is comprehensively validated using the standard dataset CoNLL-2014. The proposed model’s performance is evaluated by comparing it with baseline models in terms of accuracy, recall, F1 score, average edit distance, BLEU score, and processing time. Ablation experiments are conducted to analyze the contribution of each model component to error correction performance. The importance of the pretraining phase and the effectiveness and scalability of the dependency self-attention mechanism are also validated. (5) Conclusion: This section summarizes the significant improvements in error correction efficiency and accuracy, particularly the enhancements in accuracy, recall, and F1 score. It also highlights the advantages of the model in terms of edit distance and processing time. The limitations of the research are discussed, including language applicability, dataset coverage, and the model’s efficiency in handling long texts. Suggestions for future research directions are presented, focusing on performance optimization, multilingual adaptation, technological integration, and practical application expansion to enhance the model’s generalizability and application value. The structure of this study is organized to logically present the research approach, from theory to practice, and from model design to experimental validation, providing a complete solution for intelligent text error correction in the field.

## Model design

### BERT algorithm

BERT is a pre-trained language model based on the Transformer architecture, proposed by Google in 2018. Its pre-trains the model on large-scale unlabeled text data and then fine-tunes it on specific tasks to achieve outstanding performance. The key innovation of BERT lies in its bidirectionality, which considers the context information of each position in the sentence during pre-training, enabling the model to comprehensively consider the information of all words when understanding the sentence context. Additionally, BERT adopts the encoder structure of Transformer, which better captures long-distance dependencies and uses self-attention mechanism to handle word relationships in input sequences [16,17]. BERT has achieved remarkable results in various NLP tasks, including text classification, named entity recognition, question answering systems, etc. Its powerful language representation capability has made it a research hotspot in the field of NLP and has been widely applied in various practical applications in the industry.

### Dependency parser for erroneous sentences

The dependency parser for erroneous sentences aims to extract valid dependency information from erroneous sentences for subsequent text correction. Researchers proposed an Error-Repair Dependency Parser (ERDP) capable of correcting erroneous sentences and parsing the dependency relationships of the corrected sentences. While this parser possesses some dependency parsing capability, its error correction ability is quite limited. Since this study only aims to extract correct dependency information from erroneous sentences, an arc pruning operation is added based on its operation, proposing a Dependency Parser for Ungrammatical Sentences (DPUS) to extract effective dependency syntax information from erroneous sentences.

DPUS takes erroneous sentences as input and outputs the parsed dependency distance matrix of the sentences. Initially, $pendings=\left[p_{1},p_{2},\ldots ,p_{n}\right]$, initialized to the respective words $\left[x_{1},x_{2},\ldots ,x_{n}\right]$ of the erroneous sentence. Simultaneously, the output dependency arc set Ares is set to empty, where pendings is a list of all characters without parent nodes [18,19]. After initialization, DPUS calculates the score of each action at different positions $p_{j}$ of pendings, as shown in Equation (1) [20]:


$$score\left({action}_{i},p_{j}\right)=\vec{w}\cdot \varphi \left({action}_{i},p_{j}\right)$$ (1)

In Equation (1), ${action}_{i}$ represents selecting the i-th operation from the Actions set; $p_{j}$ denotes the j-th element in the pendings list; $\vec{w}$ is a learned weight parameter during training, and $\varphi \left({action}_{i},p_{j}\right)$ represents the feature representation of action ${action}_{i}$ at operation $p_{j}$ [21]. $\varphi \left({action}_{i},p_{j}\right)$ includes structural, unigram, bigram, and prepositional phrase attachment features. The action set Actions is defined as shown in Equation (2):


Actions={Attachleft,Attachright,Delete,Insert,Substitute} (2)

Attachleft(i)signifies $p_{i}$ becoming the child node on the right of word $p_{j}.right$, removing $p_{j}$ from pendings, with its computation outlined in Equations (3) and (4):


$$Arc.add(p_{i},p_{i}.right)$$ (3)

$$pendings.remove\left(p_{i}\right)$$ (4)

Similarly, Attachright(i) denotes $p_{i}$ becoming the child node on the left of word $p_{i}.left$, removing $p_{i}$ from pendings, as shown in Equations (5) and (6):


$$Arc.add(p_{i},p_{i}.left)$$ (5)

$$pendings.remove(p_{i})$$ (6)

In the delete operation, $p_{i}$ is an extra word, marked $p_{i}$ and removed from pendings, as demonstrated in Equations (7) and (8):


$$p_{i}.mark=delete$$ (7)

$$pendings.remove(p_{i})$$ (8)

Insert(i) inserts the word $p_{j}$ before $p_{i}$ to make the sentence smoother, simultaneously marking $p_{j}$, as illustrated in Equations (9) and (10):


$$p_{i}.mark=insert$$ (9)

$$pendings.insert\left(p_{i},location=i\right)$$ (10)

Substitute(i) replaces $p_{j}$ with a more appropriate word, as shown in Equation (11):


$$p_{i}=word$$ (11)

By comparing the scores of N-gram language models, better candidate words are found and used from their candidate word list. DPUS continuously executes the operation with the highest score until only one word remains in pendings, which becomes the root node of the dependency tree [22–24]. All arcs connected to marked words are removed to obtain the final parsing output, as demonstrated in Equations (12) and (13):


$$Arc.remove(p_{i}.hasmark,p_{j})$$ (12)

$$Arc.remove(p_{i},p_{j}.hasmark)$$ (13)

Finally, the dependency distance between every two words in the sentence is computed from the parsing result. Defining the dependency distance between the i-th and j-th words as shown in Equation (14):


$$\begin{array}{c} {Dist}_{i,j}=\left\{ \begin{array}{c} 0\;\;\;\;\;\;\;\;\;\;\;\;\;\;\;\;\;\;\;\;\;\;\;\;\;\;\;\;\;\;\;\;\;\;\;\;\;\;\;\;\;\;i=j\\ 1+\max\left(\cdot {Dist}_{i,j}\right)\;\;\;\;\;\;\;\;unreachable(i,j)\\ LeastArcs\left(i,j\right)\;\;\;\;\;\;\;\;\;\;\;\;\;\;\;\;\;\;\;\;\;\;\;\;else \end{array}\right.\; \end{array}$$ (14)

In Equation (14), unreachable(i,j) represents words i and j cannot reach each other through undirected arcs, $\max\left(\cdot {Dist}_{i,j}\right)$ indicates the maximum dependency distance among all reachable word pairs in the sentence, and LeastArcs(i,j) signifies the minimum number of undirected arcs required to reach from the i-th word to the j-th word [25–27].

### Dependency self-attention-based sequence correction model

Based on the dependency self-attention mechanism, the sequence correction model selects Transformer as the base architecture, comprising an encoder and a decoder. Replacing the self-attention mechanism in the encoder with the dependency self-attention mechanism leads to the proposal of a dependency self-attention-based sequence-to-sequence correction model. The encoder of the dependency self-attention sequence-to-sequence correction model consists of N identical stacked encoding layers [28]. Given a syntactically erroneous sentence $\left[x_{1},x_{2},\ldots ,x_{n}\right]$, its hidden state input in the l-th encoding layer is denoted as $H^{l-1}\in {\mathbb{R}}^{n\times d_{model}}$, where $d_{model}$ is the model’s dimension, and n is the length of the sentence. The computation of the encoding layer is outlined in Equations (15)‒(17):


$$Z^{l}=MultiHead(H^{l-1})$$ (15)

$$M^{l}=LayerNorm(H^{l-1}+Z^{l})$$ (16)

$$H^{l}=LayerNorm(M^{l}+FFM(M^{l}))$$ (17)

In equations (15)‒(17), $H^{l}$ represents the hidden state output of layer i, MultiHead denotes the multi-head self-attention mechanism, LayerNorm stands for layer normalization, and FFM refers to the feed-forward neural network. MultiHead consists of multiple self-attention heads, and its computation is illustrated in Equation (18):


$$MultiHead\left(H^{l-1}\right)=Concat({head}_{1}^{l};{head}_{2}^{l};\ldots ;{head}_{n}^{l})W_{mul}^{l}$$ (18)

In Equation (18), $W_{mul}^{l}$ represents trainable parameters.

The dependency self-attention mechanism is introduced by incorporating the sentence’s dependency distance information into the attention calculation of each individual head, as depicted in Equation (19):


$${head}_{i}^{l}=softmax(\frac{D^{h}⨀Q_{i}^{l}{\left(K_{i}^{l}\right)}^{T}}{\sqrt{d}})V_{i}^{l}$$ (19)

In Equation (19), $D^{h}\in {\mathbb{R}}^{T\cdot T}$ represents the dependency matrix of the sentence, as shown in Equation (20):


$$D_{i,j}^{h}=\alpha^{{Dist}_{i,j}}$$ (20)

In Equation (19), ${Dist}_{i,j}$ represents the dependency distance between the ith word and the jth word, where α is a hyperparameter ranging from 0 to 1, and ⨀ denotes the Hadamard product.

The decoder, similar to the encoder, consists of N decoding layers. It receives the final layer’s output hidden state from the encoder and decodes it to generate the corrected sentence output [29]. Each decoding layer includes multi-head self-attention module, feed-forward neural network, and encoder-decoder attention mechanism. The difference between the encoder-decoder attention mechanism and the self-attention module lies in that the former takes input from the encoder’s hidden state, while the latter takes input from its own hidden state [30–32].

Assuming the hidden state output by the decoder’s final layer at step i is $Z_{i}$, after passing through a fully connected layer and then through the softmax function, the probability distribution of the decoded character output at step i on the vocabulary table can be obtained, as shown in Equation (21):


$$P_{vocab}=softmax(W_{d}Z_{i}+b_{d})$$ (21)

In Equation (21), $W_{d}$ and $b_{d}$ are trainable parameters.

### Overall model architecture

**Fig 1:**
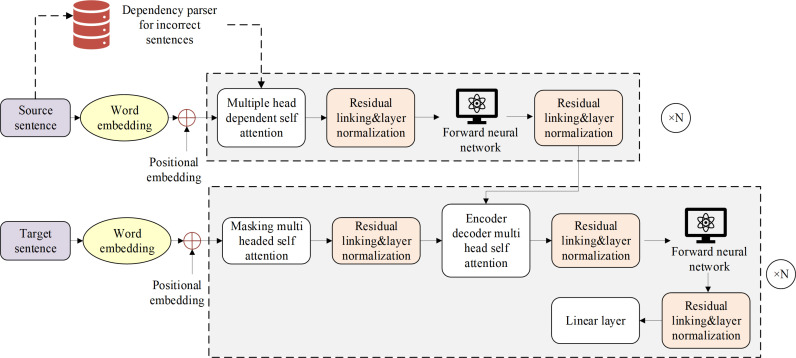
Overall Model Architecture.

The overall architecture of the model is shown in Fig 1.

The model consists of two parts: a dependency parser and a sequence-to-sequence correction model based on dependency self-attention. Firstly, the dependency parser, which is specifically designed for erroneous sentences, is used to analyze the sentences and produce dependency structure outputs [33,34]. Then, the dependency distance is calculated based on the dependency results. Finally, the dependency distance is incorporated into the self-attention computation of the sequence-to-sequence correction model, adjusting the attention weights according to the dependency distance to build a sequence-to-sequence correction model based on dependency self-attention. Additionally, this study introduces a pretraining phase, which enhances the model’s correction performance through pretraining and fine-tuning.

## Experimental data design

This study uses the CoNLL-2014 dataset [35]. The CoNLL-2014 dataset uses the preprocessed Nucle dataset as the training set, while the test set includes 50 essays written by non-native English-speaking students. Each sentence in the test set is corrected by two experts, and the type of each error in the sentence is also annotated by the experts. All experiments are conducted on a server equipped with an NVIDIA A100 Graphics Processing Unit (GPU). The operating system is Ubuntu 20.04, and the framework used is PyTorch version 1.10.0. All model training and inference are executed in this environment, with each training epoch taking approximately 2.7 hours.

This study selects the BERT algorithm as the basic framework. In the data preprocessing stage, SpaCy tool is used to regularize the sentence format, and Byte Pair Encoding (BPE) is used to tokenize the sentences, with a dictionary size of 8000. In the back-translation stage, Transformer (Big Setting) is used as the model architecture. In addition to adding noise to the vocabulary during inference, character perturbations are added to the generated sentences by adding, deleting, or swapping characters to introduce word spelling errors. In the pre-training and fine-tuning stages, a sequence-to-sequence text correction model with dependency self-attention mechanism is used, with model structure parameters consistent with Transformer (Big Setting). In the pre-training stage, Label Smoothed Cross Entropy (LSCE) is used as the loss function, with a smoothing coefficient set to 0.1. Gradient clipping is applied to gradients greater than 1, and Adam is used as the optimizer, with $\beta_{1}$ set to 0.9, $\beta_{2}$ set to 0.998, and ε set to 1 × 10^−8^. In the fine-tuning stage, the Adafactor optimizer is used, with a maximum character count of 4096 per batch. The model is pre-trained for 10 epochs and fine-tuned for 20 epochs. In the dependency self-attention mechanism, the decay coefficient is set to 0.965, and the dependency self-attention mechanism is used on 6 attention heads. If a character is split into multiple parts by BPE encoding, each part inherits the dependency distance relationship of the original word. During decoding, Beam Search is used to enhance the model’s correction ability, with a Beam Size of 5 on the CoNLL-2014 dataset. The parameter settings are shown in Table 2.

**Table 2:** Parameter Settings.

Experimental setup	Parameter settings
Model architecture	Transformer (Big Setting)
dictionary size	8000
loss function	label smoothing cross entropy
Smoothing coefficient	0.1
optimizer	Adam
$\beta_{1}$	0.9
$\beta_{2}$	0.998
ε	1 × 10^-8^
Maximum number of characters	4096
Number of training epochs	Pre-training 10 epochs, fine-tuning 20 epochs
Attenuation coefficient	0.965
Number of attention heads	6
Beam Size	5

The baseline Transformer model configuration is shown in Table 3.

**Table 3:** Baseline Transformer model configuration.

Configuration items	Baseline Transformer model configuration
Model architecture	Transformer Encoder-Decoder Architecture
Number of layers	Encoder layers: 6 layers; Decoder layers: 6 layers
Hidden layer size	512 hidden units
Number of attention heads	8 attention heads
Feedforward network size	2048 hidden units
Position encoding	Sine-cosine position encoding
Optimization algorithm	Adam optimizer
Learning rate	Initial learning rate: 0.0001; Learning rate decay: linear decay
Training cycle	20 training epochs
Batch size	64
Gradient clipping	Maximum gradient clipping value: 1.0

## Experimental results and analysis

### Comparison on the CoNLL-2014 dataset

**Fig 2:**
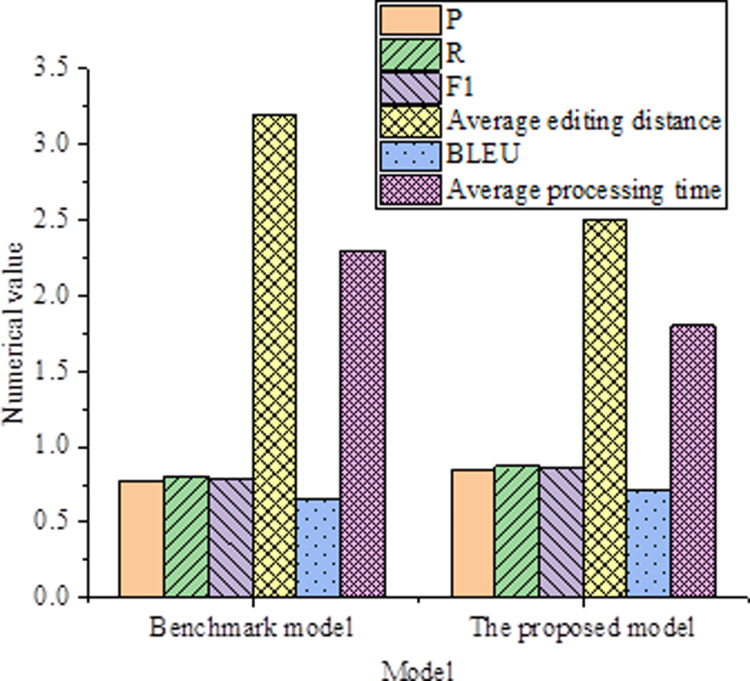
Comparison Results on CoNLL-2014 Dataset.

The performance comparison between the baseline model and the model proposed in this study on different metrics is shown in Table 4 and Fig 2.

**Table 4:** Performance Comparison Results of the Baseline Model and the Model Proposed in This Study on Different Metrics.

Index	Baseline model	This study model
Precision (P)	0.78	0.85
Recall (R)	0.81	0.87
F1-value	0.79	0.86
average edit distance	3.2	2.5
BLEU score [36]	0.65	0.72
Average processing time (seconds)	2.3	1.8

The data results indicate that the proposed model outperforms the baseline model in metrics such as accuracy, recall, F1 score, average edit distance, BLEU score, and average processing time. The accuracy of the proposed model is 0.85, slightly higher than the baseline model’s 0.78. In terms of recall, our model achieves 0.87, while the baseline model is at 0.81. The F1 score, which is the harmonic mean of precision and recall, is 0.86 for our model, higher than the baseline model’s 0.79. This suggests that our model can better maintain a balance between precision and recall in the correction task. The average edit distance measures the difference between two strings, with the proposed model having an average edit distance of 2.5, smaller than the baseline model’s 3.2. This implies that the proposed model can more effectively convert erroneous sentences into correct ones, with a smaller edit distance. The BLEU score, a commonly used metric in machine translation, indicates the similarity between generated sentences and reference sentences. The proposed model’s BLEU score is 0.72, higher than the baseline model’s 0.65, indicating that the proposed model’s generated sentences are closer to the reference sentences. The average processing time of our model is 1.8 seconds, shorter than the baseline model’s 2.3 seconds. This indicates that the proposed model is more efficient in the correction task, able to process input data more quickly. The proposed model demonstrates superiority in various metrics, with higher accuracy, efficiency, and performance, making it suitable for text correction tasks.

**Fig 3:**
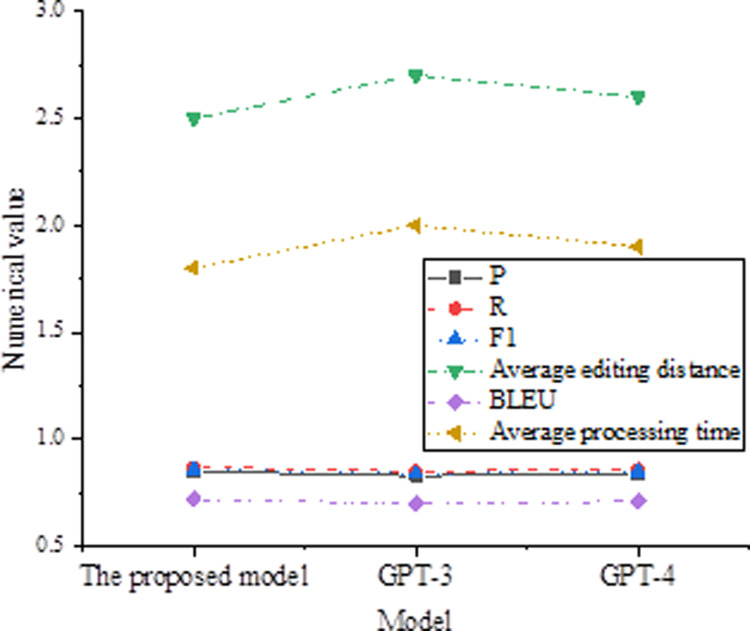
Comparison with Advanced Models.

To further validate the effectiveness and advancement of the proposed model, it is compared with the current state-of-the-art models, including GPT-3 and GPT-4. These models have made significant strides in NLP tasks, particularly excelling in text generation and error correction tasks. Experiments are conducted on the CoNLL-2014 dataset, and the comparison results are shown in Fig 3.

In Fig 3, although the GPT-3 and GPT-4 models excel in certain metrics, the proposed model demonstrates superior performance in key indicators such as accuracy, recall, and F1 score. Specifically, the proposed model achieves an accuracy of 0.85, a recall of 0.87, and an F1 score of 0.86, all surpassing the performance of GPT-3 and GPT-4 models. Additionally, the proposed model has an average edit distance of 2.5, which is lower than GPT-3’s 2.7 and GPT-4’s 2.6, indicating that the proposed model is more effective at converting erroneous sentences into correct ones. In terms of BLEU scores, the proposed model scores 0.72, higher than GPT-3’s 0.70 and GPT-4’s 0.71, suggesting that sentences generated by the proposed model are more similar to the reference sentences. Moreover, the proposed model has an average processing time of 1.8 seconds, which is lower than GPT-3’s 2.0 seconds and GPT-4’s 1.9 seconds, indicating greater efficiency in error correction tasks. These results demonstrate that the proposed model performs exceptionally well in text correction tasks, offering high accuracy, efficiency, and performance, making it suitable for practical applications. Despite the advantages of large language models like GPT-3 and GPT-4 in NLP, the proposed model provides an efficient and high-performance solution in resource-constrained environments. Overall, the proposed model shows superiority across various metrics and is well-suited for text correction tasks. Future research could explore further integration with large language models to potentially enhance performance and efficiency in text correction tasks.

### Analysis of ablation experiment results

**Fig 4:**
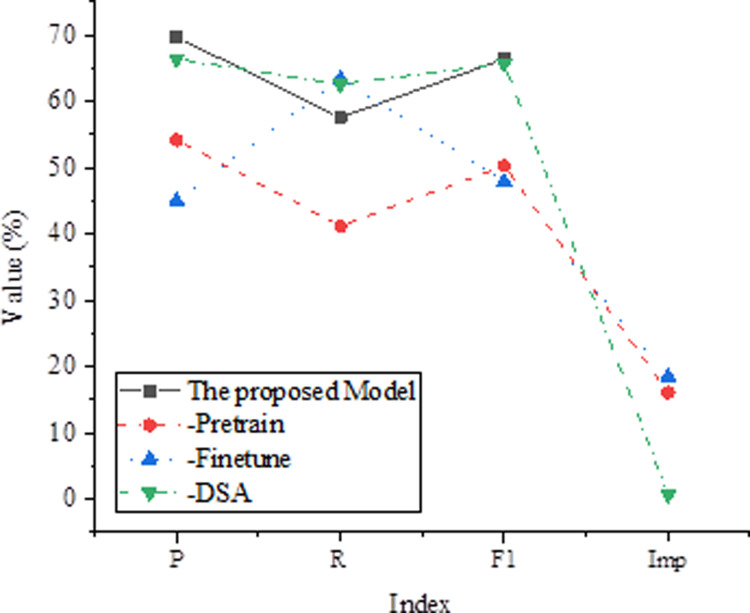
Ablation Experiment Results.

Ablation experiments aim to verify the impact of each part of the model on the text correction task. In this study, ablation experiments are conducted on the CoNLL-2014 dataset, gradually removing different parts of the model and evaluating their impact on correction performance. “-Pretrain” represents the performance of the model without removing the pretraining phase. “-Finetune” represents the performance of the model without the fine-tuning phase. “-DSA (Dependency Self-Attention)” represents the performance of the model without the dependency self-attention mechanism. “Imp” indicates the performance change in F1 score after removing the corresponding module. The results of the ablation experiments are shown in Fig 4.

The experimental results show that the proposed model performs best when the pretraining phase is retained, with precision (P) of 69.7%, recall (R) of 57.6%, and an F1 score of 66.4%. However, when the fine-tuning phase is removed, the model’s performance significantly decreases, with precision dropping to 54.2%, recall dropping to 41.2%, and the F1 score dropping to 50.3%. Additionally, if the dependency self-attention mechanism is removed, the model’s performance slightly decreases, but the impact is not significant, with only a 0.7% decrease in the F1 score. These results indicate that the pretraining and fine-tuning phases are crucial for the model’s performance, while the dependency self-attention mechanism has a minor impact on performance. These findings have important implications for the design and optimization of text correction models.

### Effectiveness of dependency self-attention mechanism

To validate the effectiveness and scalability of the dependency self-attention mechanism, this study conducts verification experiments on the CoNLL-2014 dataset. Parameter optimization and structural adjustments are made to enhance the dependency self-attention mechanism, including fine-tuning the weights of dependency relationships, introducing more refined methods for calculating dependency distances, and performing extensive testing across various tasks and datasets. The representation of each model is shown in Table 5.

**Table 5:** Meaning of Each Model.

Models	Description
Model	Proposed sequence-to-sequence text error correction model with dependency self-attention
Model-DSA	Remove the self-attention mechanism based on dependency distance in the model
Model/DSA-L	Replace the DPS (Dependency Parser) with a normal dependency parser
Model/-Pretrain	Remove pre-training based on the model in this article
Model/-Pretrain-DSA	Remove pre-training and dependency self-attention mechanism based on the model
Model+BERT	Add the BERT Fusion module to the model in this article, and replace the encoder’s self-attention with the dependency self-attention module
Model+BERT/-DSA	Delete the dependency self-attention mechanism in the Model+BERT model
Optimized Model+BERT	Model+BERT model after optimizing the dependency self-attention mechanism

**Fig 5:**
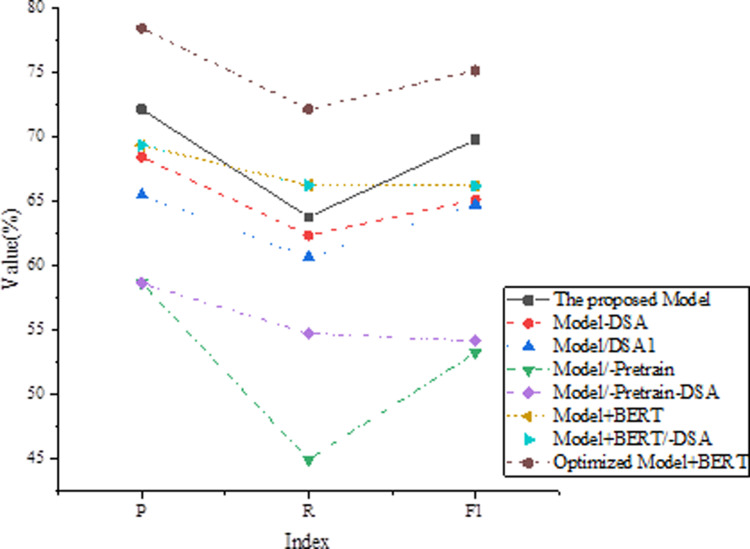
The Effectiveness Verification Experiment of Dependency Self-Attention Mechanism.

The experimental results of the effectiveness verification of the dependent self-attention mechanism are shown in Fig 5.

Fig 5 displays the performance of different models in terms of Precision (P), Recall (R), and F1 score. The proposed model shows higher P (72.11%), R (63.77%), and F1 (69.76%). Removing the dependency self-attention mechanism results in a decline in performance for Model-DSA, with P dropping to 68.43%, R to 62.34%, and F1 to 65.12%. Model/DSA1, which replaces the dependency self-attention mechanism, shows P at 65.46%, R at 60.64%, and F1 at 64.73%. Excluding the pre-training phase in Model/-Pretrain results in lower P (58.64%) and R (44.98%), with F1 significantly dropping to 53.25%. Model/-Pretrain-DSA, which omits both the pre-training phase and the dependency self-attention mechanism, also performs poorly with P and R at 58.64% and F1 at 54.17%. Introducing the BERT module in Model+BERT improves P to 69.34%, R to 66.25%, and F1 to 66.19%. Removing the dependency self-attention mechanism from Model+BERT, resulting in Model+BERT/-DSA, maintains P at 69.34%, but R slightly decreases to 66.23% and F1 to 66.17%. Finally, the optimized Model+BERT demonstrates the best performance with P (78.4%), R (72.12%), and F1 (75.13%), highlighting the model’s superiority in text intelligent correction tasks. Overall, the experimental results clearly indicate that different model configurations significantly impact performance in text correction tasks. Removing the pre-training phase leads to a substantial performance decline, while incorporating the BERT Fusion module effectively enhances performance. Furthermore, the optimization effect of the dependency self-attention mechanism in the model is validated, as its removal affects model performance.

### Qualitative analysis

To validate the practical effectiveness of the model, Table 6 presents qualitative examples of corrected sentences.

**Table 6:** Qualitative Examples of Corrected Sentences.

Original sentence	Wrong sentence	Corrected sentence	Qualitative analysis
The boy who quickly ran to the store forgot to buy bread.	The boy who quickly ran to the store forget to buy bread.	The boy who quickly ran to the store forgot to buy bread.	The verb tense was wrong, and the model successfully corrected forget to forgot.
Although it was raining, they decided to go for a walk.	Although it was raining, they decided to goes for a walk.	Although it was raining, they decided to go for a walk.	The verb form was wrong, and the model successfully corrected goes to go.
She found the book that she had lost last week.	She finded the book that she had lost last week.	She found the book that she had lost last week.	The verb past tense was wrong, and the model successfully corrected founded to found.
The committee, which includes several experts, is meeting tomorrow.	The committee, which includes several experts, are meeting tomorrow.	The committee, which includes several experts, is meeting tomorrow.	The subject-verb agreement was wrong, and the model successfully corrected are to is.
Despite the challenges, they have managed to maintain their progress.	Despite the challenges, they have manage to maintain their progress.	Despite the challenges, they have managed to maintain their progress.	The verb form was wrong, and the model successfully corrected manage to managed.

These cases demonstrate that the proposed model excels in handling complex sentence structures and grammatical errors, effectively improving the accuracy and efficiency of text intelligent correction.

## Conclusion

This study proposes an integrated model that combines the dependency self-attention mechanism, aimed at improving the efficiency and accuracy of intelligent text correction in English translation. Firstly, the BERT algorithm is chosen as the foundational framework, improved upon, and validated through experiments on the CoNLL-2014 dataset. This dataset, widely used in linguistic research, contains a diverse range of grammatical error samples. By comparing the performance of the baseline model and the proposed model across various metrics, the model excels in accuracy, recall, F1 score, average edit distance, BLEU score, and average processing time. Specifically, the proposed model improves accuracy from 0.78 to 0.85, recall from 0.81 to 0.87, and F1 score from 0.79 to 0.86 compared to the baseline model. Additionally, the proposed model significantly improves the average edit distance from 3.2 to 2.5 and increases the BLEU score from 0.65 to 0.72. In terms of processing time, the proposed model reduces the average processing time from 2.3 seconds to 1.8 seconds compared to the baseline model.

Furthermore, this study conducts ablation experiments to validate the impact of each component in the model on the text correction task. The results indicate that the pre-training stage is crucial for model performance, while the dependency self-attention mechanism has a minor impact. Specifically, when the fine-tuning stage is removed, the model’s performance significantly drops, with accuracy decreasing to 54.2%, recall to 41.2%, and F1 score to 50.3%. Additionally, removing the dependency self-attention mechanism results in a slight performance decrease, with only a 0.7% decrease in F1 score. These results suggest that the pre-training and fine-tuning stages are critical for model performance, while the dependency self-attention mechanism has a minor impact. Finally, the effectiveness and scalability of the dependency self-attention mechanism are validated. Experimental results demonstrate that under different configurations, the dependency self-attention mechanism effectively improves model performance.

The integrated model proposed in this study performs admirably in the task of intelligent text correction in English translation, providing an effective solution to enhance correction efficiency and accuracy. It offers new methods and insights for the field of intelligent text correction, which is crucial for improving the effectiveness of NLP technology in practical applications. Despite achieving certain results, there are limitations that need to be addressed. Firstly, the study primarily focuses on intelligent text correction tasks in English translation, and further exploration is needed to assess its applicability to other languages or domains. Secondly, although the experiments are validated using the CoNLL-2014 dataset, it may not cover samples from all languages and grammatical structures, thus requiring further verification of the model’s generalization ability. Additionally, the model may face efficiency issues when handling long texts since the current focus is mainly on sentence-level correction, potentially leading to subpar performance for errors at the discourse level. Future studies can be expanded in several directions. Firstly, there’s room to further optimize the model’s performance, especially concerning efficiency and accuracy when dealing with long texts and complex grammatical structures. Secondly, exploring the design of multi-language intelligent text correction models to cater to different language environments is essential. Furthermore, integrating other techniques such as reinforcement learning and transfer learning could further enhance the model’s performance and generalization ability. Lastly, applying the model to broader fields like automatic text editing and machine translation to explore its effectiveness and value in practical applications.

## Supporting information

S1 Data This compressed file contains supplementary materials used in the research paper, including: detailed descriptions of algorithm implementations; source code for model training and testing; experimental datasets; and high-resolution image files for Figs 2–5 in the article.
(RAR)

S2 Data This Excel file contains raw numerical data for the experimental results, including performance metrics, comparative experimental results, and related experimental variables.
(XLSX)
